# Coffee-Associated Endophytes: Plant Growth Promotion and Crop Protection

**DOI:** 10.3390/biology12070911

**Published:** 2023-06-25

**Authors:** Suhail Asad, Alviti Kankanamalage Hasith Priyashantha, Saowaluck Tibpromma, Yinling Luo, Jianqiang Zhang, Zhuqing Fan, Likun Zhao, Ke Shen, Chen Niu, Li Lu, Itthayakorn Promputtha, Samantha C. Karunarathna

**Affiliations:** 1 School of Biology and Chemistry, Pu’er University, Pu’er 665000, China; luoyl1978@foxmail.com (Y.L.); drjqzhang@126.com (J.Z.); fanzhuqing1122@gmail.com (Z.F.); zhaozejin126@126.com (L.Z.); shanke11223@gmail.com (K.S.); 2 Independent Researcher, Gampaha District, Nittambuwa 11880, Sri Lanka; priyashanthahasith@gmail.com; 3 Center for Yunnan Plateau Biological Resources Protection and Utilization, College of Biological Resource and Food Engineering, Qujing Normal University, Qujing 655011, China; saowaluckfai@gmail.com (S.T.); 6371105004@lamduan.mfu.ac.th (L.L.); 4 Spice and Beverage Research Institute, Chinese Academy of Tropical Agriculture Sciences, Haikou 570100, China; niuchen1123@gmail.com; 5 Department of Biology, Faculty of Science, Chiang Mai University, Chiang Mai 50200, Thailand; itthayakorn.p@cmu.ac.th; 6 Environmental Science Research Center, Faculty of Science, Chiang Mai University, Chiang Mai 50200, Thailand; 7 National Institute of Fundamental Studies (NIFS), Hantana Road, Kandy 20000, Sri Lanka

**Keywords:** ascomycetes, bio decaffeination, endophytic bacteria, endophytic fungi, entomopathogenic endophytes, nitrogen fixing, phosphate solubilizing, phytohormones, plant defense system

## Abstract

**Simple Summary:**

Several hundreds of endophytic microbes are found to be associated with coffee plants. Endophytes are mostly recognized as symbionts and offer many benefits to plants. Several studies in different laboratories and natural environments show that endophytes are responsible for enhancing coffee plant growth and minimizing various pest damages. This shows that coffee endophytes can be sustainable alternatives to chemical pesticides. In the coffee beverage industry, using coffee endophytes is more advantageous as they improve the aroma and flavor of the beverage. Limited studies have been carried out in this regard; nevertheless, in this article, we highlighted the importance of coffee endophytes based on the available study findings.

**Abstract:**

Endophytic microbes are a ubiquitous group of plant-associated communities that colonize the intercellular or intracellular host tissues while providing numerous beneficial effects to the plants. All the plant species are thought to be associated with endophytes, majorly constituted with bacteria and fungi. During the last two decades, there has been a considerable movement toward the study of endophytes associated with coffee plants. In this review, the main consideration is given to address the coffee-associated endophytic bacteria and fungi, particularly their action on plant growth promotion and the biocontrol of pests. In addition, we sought to identify and analyze the gaps in the available research. Additionally, the potential of endophytes to improve the quality of coffee seeds is briefly discussed. Even though there are limited studies on the subject, the potentiality of coffee endophytes in plant growth promotion through enhancing nitrogen fixation, availability of minerals, nutrient absorption, secretion of phytohormones, and other bioactive metabolites has been well recognized. Further, the antagonistic effect against various coffee pathogenic bacteria, fungi, nematodes, and also insect pests leads to the protection of the crop. Furthermore, it is recognized that endophytes enhance the sensory characteristics of coffee as a new field of study.

## 1. Introduction

### 1.1. Coffee as One of the Most Appealing Beverages 

The genus coffee belongs to the Rubiaceae family. There are about 103 coffee species available, though, Arabica (*Coffea arabica*) and Robusta (*Coffea canephora*) are the two most dominant coffee species that grow worldwide and also provide about 99% of the global market [1,2]. Coffee is also recognized as the second most traded commodity worldwide after oil. According to the International Coffee Organization [3], world coffee exports amounted to 10.88 million (60-kilo) bags in December 2022, while exports in the first 3 months of the coffee year 2022/2023 (22 October to 22 December) were 30.27 million (60-k) bags. The coffee market is also rapidly expanding worldwide; for example, in China, coffee consumption has grown steadily by 20% each year, particularly in urban areas [4].

Moreover, as of today, coffee plants are grown in over 60 countries by more than 25 million farmers, particularly small-scale growers in Africa, Americas and Asia [1]. In fact, those small-scale farmers dominate the market—95% of coffee farmlands are 5 ha or smaller, and 84% are under 2 ha. The remaining 5% is produced by larger estates, and coffee plantations larger than 50 ha are rare outside Central and South America [5]. For instance, in Ethiopia, coffee is the top export, making between 20–25% of all foreign exchange earnings, and around 15 million people are thought to depend on coffee for their livelihood; more than 80% of coffee growers are peasant farmers [6]. 

### 1.2. Concerns of Coffee Harvest Reduction Factors and Preventive Strategies 

Despite the fact that coffee has a significant economic impact on low-income countries throughout the world, the sector has been faced with numerous persistent stresses and unexpected shocks over the decades challenging productivity. These common issues include climatic changes, diseases, and pests [7,8]. Coffee plantations are more susceptible to climate changes, such as extreme temperature (low or high temperature), variable precipitation patterns (drought and waterlogging), and elevated atmospheric carbon dioxide (CO_2_) concentrations. The plants are also sensitive to other environmental conditions such as alteration of soil pH and pollutants. These conditions have direct effects on coffee productivity, and also indirect effects by increasing disease severity, and lowering the nutritional quality of coffee. Overall, climatic changes alone could contribute up to 70% of the coffee yield reduction [8].

Addressing the issues of diseases, coffee rust caused by the fungus *Hemileia vastatrix* is among the leading troublesome organisms, and the first known outbreak was documented in Sri Lanka in 1869. The thunderdome of the epidemic led Sri Lanka to replace their coffee lands with tea and other alternative crops. Subsequently, the disease affected nearly all regions of the world where coffee was grown [9]. In extreme outbreaks, foliage can be lost up to 100% and berries can be lost up to 70%, and overall the disease can result in yield losses of more than 75% [10]. Yet, the disease is not eradicated and is responsible for causing losses of one to two billion US dollars annually [11]. In addition, a lack of awareness and surveillance about the rust disease (also others) could be a future alarming threat to emerging coffee-grown countries such as China [12]. Other prominent diseases include the coffee berry disease caused by *Colletotrichum kahawae* [13], coffee wilt disease caused by *Fusarium xylarioides* [14], brown eye spot caused by *Cercospora coffeicola* [15], and Armillaria Root Rot caused by *Armillaria mellea* [16]. In turn, coffee berry borer *Hypothenemus hampei* [17], coffee leaf miner *Leucoptera coffeella* [18], and black coffee twig borer *Xylosandrus compactus* [19] are among the major insect pests of coffee. Despite those, plant parasitic nematodes also severely hamper coffee plantations in many parts of the world [20]. The management of those disease and pest issues is generally achieved by synthetic pesticides, however, such practices are deemed to be negligible by many communities. At the same time, other available traditional techniques such as shade management, bark scrubbing, stem wrapping to prevent oviposition, mass trapping with the aid of pheromone-baited traps, and uprooting and burning of affected plants are not expected to control the pest trouble fully [21,22].

### 1.3. Endophytes as a Prime Source for Improving Plant Health

Recently, endophytes have been recognized as prime candidates for protecting crops from both abiotic and biotic stresses, thus becoming a hot-topic [23,24]. Simply, endophytes can be described as any organism that lives within plant tissues. Interestingly, throughout their life cycles, endophytic communities are closely related to their hosts or spend portions of their lives in host tissues without causing disease or negatively affecting them [25]. Thus, endophytic association generally shows a mutualistic relationship, with some exceptions [26]. 

Theoretically, it is accepted that almost all plants are infested with endophytes, at least one or more of them [27]. A plethora of studies have been conducted on many different forms of endophytic populations, including bacteria and fungi, regarding their various potential benefits to plants. Those include producing growth-promoting metabolites, insect and pest repellents, antimicrobials to fight plant pathogens, and protecting plants in stressful environments [27,28,29]. The majority of the studies showed that endophytes isolated from the same plants may have the ability to promote plant growth. Several research studies have documented how endophytic bacteria can encourage the growth of non-host plants [30]. 

Other than coffee, studies have found that these endophyte microorganisms harbor many other crop plants including chickpea, chili, citrus, cotton, cowpea, maize, mustard, pearl millet, rice, soybean, strawberry, sugarcane, sunflower, tomato, and wheat [31,32,33]. Endophytes can also be found in below or above-ground parts of these plants, e.g., roots, tubers, barks, flowers, fruits, leaves, nodules, ovules, petioles, seeds, stems, and twigs [34,35]. 

### 1.4. Aim of the Current Article 

In the above context, we reviewed the studies of endophytic bacteria and fungi associated with coffee plants. The main purpose of this paper is three-fold; (1) to emphasize the endophytes that possibly influence coffee plant growth promotions, (2) to report the potentiality of coffee endophytes in protecting coffee plants against various plant pathogens and insect pests, and (3) to understand the scenario on the available studies, gaps, and future directions. In addition to these major considerations, we briefly discussed the ability of endophytes to improve the quality and flavor of coffee seeds. To obtain the necessary information, in this article, we analyzed 107 studies published in 50 research papers. The data extraction pipeline is shown in Figure S1.

## 2. General Aspects of Endophytes

### 2.1. Endophytic Association with Plants

Endophytes are said to be universally distributed within the plant kingdom, and their presence in land, freshwater, and marine plants has been recorded [36,37]. Nonetheless, they can be latent pathogens, mutualists, commensals, temporary residents, or latent saprotrophs [38]. The majority of plant endophytes are found to be bacteria and fungi, while archaebacteria, algae, protozoa, viruses, and nematodes are rare endophytes [39]. According to modern molecular-based techniques and fossil records, the co-evaluation of endophytes has been reported to be from about 460 million years along with the plants [40,41]. This initial association is probably with the arbuscular mycorrhizal fungi; based on the sequence analyses, it was further revealed that the development of those endophytes was along with the land plant colonization [42]. According to the preservations in the Rhynie chert, this association runs to the period of Early Devonian, where the plant lacks a ‘true’ root system [43]. The absence of well-preserved leaf endophyte fossils prompts the hypothesis that they likely originated later, during the Early Cretaceous period, concurrent with the advent and diversification of angiosperms. In contrast, studies show that fungus leaf endophytes existed at least during the Carboniferous period [44]. A study of leaf endophytes in the coffee family (Rubiaceae) conducted by Verstraete et al. [45] revealed that plant lineages with endophytes show a higher level of speciation, which is also significant compared with the plant lineages without endophytes, however, there is a small disparity in the extinction rate. Furthermore, plants evolve twice as quickly toward endophytes than they do away from them, proving that the interaction may benefit the plant [45].

It is relatively unusual for a plant to be colonized by just one kind of endophyte. Instead, a number of endophytes habited with the plant also probably directly or indirectly interacting with each other, e.g., bacteria–bacteria, fungi–fungi, and bacteria–fungi [46]. Thus, many millions of species of endophytes are thought to be interacting with plants [47]. Despite the fact that bacteria and fungi are completely different forms of life as they are prokaryotes and eukaryotes, respectively, they share similar characteristics as endophytes, e.g., the lifestyles of bacteria and fungi include intra- or intercellular colonization of plant tissues [48,49]. Furthermore, based on their life strategies, endophytic communities that may be present in the host plant, have either obligate or facultative associations. The obligatory endophytes can grow and survive only on the host plant; transmission through vertically (seeds) or vectors (pollinators, arthropods, or sap-feeders), rather than emanating from the rhizosphere. Unlike obligate endophytes, facultative endophytes do not show the absolute necessity of a host plant. They have a stage in their life cycles where they exist independently of their hosts and are generally associated with the soil environment and atmosphere that is in close proximity to the plant and colonize the plant once the opportunity allows via coordinated infection [50,51]. In order to create an association with the plant, for example, soil-dwelling endophytes should first colonize the rhizosphere. Plant roots secrete exudates that contain various components, mainly sugars, amino acids, organic acids, vitamins, and high molecular weight polymers, thus providing various nutritional resources to the microbes. Those exudates are concentrated in the plant rhizosphere, thereby many microbes including endophytes attract (chemotaxis) and colonize [52]. Nevertheless, it is not an easy task to colonize the rhizosphere as endophytes need to fight for space and nutrients along with other wealthy microbiota, which perhaps even seems to be antagonistic against endophytic colonization [30]. In turn, endophytes may outcompete the other microbes by growing aggressively and taking space more rapidly, however, little is understood about how they interact with other microorganisms [53,54]. The endophytes which thrive in this microenvironment could colonize further and reach the root system of the plants. In the case of bacteria, their traits including motility and the synthesis of polysaccharides are crucial for colonizing plant rhizospheres [30,55]. Further, bacterial populations use long strings of closely associated cells to get in contact with the root system [56]. More precisely, endophytes enter the plant by using two different mechanisms, namely, active and passive. In active colonization, endophytes enter plant tissue by searching and through cracks (e.g., open sites at the lateral roots emerging, areas of elongation and differentiation of the root), wounds (e.g., insect bites), or hydathodes. Moreover, in the active mechanism, they penetrate by degrading plant tissues with the help of hydrolytic enzymes such as cellulase and pectinase. The colonization of the plant by passive endophytes occurs due to random circumstances, rather than on purpose. Due to the absence of the cellular machinery needed for plant colonization, the passive life strategy may make them less competitive [51,57]. Note that endophytes that are transmitted via seeds ensure their presence in young plants. Similarly, the abovementioned procedure is unnecessary for plants that reproduce vegetatively [34].

It is possible for bacterial endophytes to colonize a plant host systematically even after a single entry. Further, like in rhizosphere colonization, bacterial endophytes use specific traits known as colonization traits for the entire plant colonization including communication between the bacterial endophytes and the plant [58]. In one study, Huang et al. [59] observed that endophytic colonization also leads the physiological changes in the plant, and according to their study, the *Bacillus subtilis* invasion started from the root hair by swelling, and the bending of root hair tips also caused dichotomous branching of the root hair without bending. Turning back to the fungi, when the spores are attached to the host surface, they start to germinate, by slight swelling, followed by germ tube emergence, development of aspersoria, penetration peg emergence, hyphae development, and invasion of plant tissues [60]. It is worth noting that a comparatively small fraction of endophytes is colonizing the above-ground part of the plant due to the restriction for nutrients acquisition, ultraviolet light (UV), and desiccation, thus the greatest number of endophytes are associated with the roots [61,62]. Unlike fungi, bacterial endophytes favor the pathway of colonizing via the xylem vascular system and therefore can find systemic colonization of internal plant compartments. It has been demonstrated that bacteria can colonize xylem vessels, and the perforation plates’ holes are big enough to let bacteria pass through [63]. Nevertheless, all the endophytes must endure the plant defense mechanism prior to colonization. Plants detect microbial invaders through microbe-associated molecular patterns (MAMPs), and through a series of defense responses, they attempt to overcome the threat [63]. Endophytes have the ability to counteract the stimulation of the plant defense response, either synthesizing MAMPs with poor elicit capacity or downregulating their expression. Further, they can even synthesize the required genes to minimize the stimulation of plant defense reactions [64]. Production of reactive oxygen species (superoxide, hydroperoxyl radical, hydrogen peroxide, hydroxyl radical species), nitric oxide, and phytoalexins are examples of plant defense reactions. In order to overcome this oxidative stress, for instance, *Enterobacter* sp. encodes superoxide dismutases, catalases, hydroperoxide reductases, chloroperoxidase, and thiol peroxidases [65]. It is also recognized that pre-communication between the endophytes and the plant is needed, which depends on the mutual signal exchange between them and also leads to balanced antagonisms [66]. Moreover, in general, owing to the selective pressure driven by the plant, the colonization of endophytes is organ- and tissue-specific [67]. 

### 2.2. Brief Classification and Important Genera/Phylogenetic Placement

Endophytic bacteria fall within 16 phyla with more than 200 genera, the majority of them belonging to three phyla, namely, Actinobacteria, Firmicutes, and Proteobacteria [68]. Endophytic bacteria are considered a subclass of rhizospheric bacteria, which are also known as plant growth-promoting rhizobacteria [30]. They also could be either Gram-negative or Gram-positive [68]. However, endophytic fungi predominantly categorize under the Ascomycetes, appearing sporadically in Basidiomycetes and Zygomycetes [29]. More precisely, endophytic fungi are classified into two main groups, (1) clavicipitaceous, which infects some grasses thus having a narrow host range; and (2) nonclavicipitaceous, which infects vascular and non-vascular plants. Overall, the species belonging to those two groups are classified into classes 1–4. Class 1 is categorized under the clavicipitaceous and other classes are placed under non-clavicipitaceous. Class 1 fungi (Ascomycota) colonize the plant’s shoots and rhizomes and Class 2 (in Dikaryomycota–Ascomycota in major and minor in Basidiomycota). Most belong to the Ascomycota, with a minority belonging to Basidiomycota. Endophytes colonize in shoots, roots, and rhizomes. Class 3 (mostly in Dikaryomycota–Ascomycota where they are especially concentrated or, Basidiomycota) and Class 4 (primarily, Ascomycota) colonize in the shoot and root, respectively. Except for Class 3, fungi in other classes show extensive plant colonization [69]. In a recent literature review by Lugtenberg et al. [70], suggested updating this classification as it hides the group of entomopathogenic fungi, which are symptomless endophytes of plants.

## 3. Coffee Endophytes: Insight

### 3.1. History of Studies, Current Trends, and Gaps

**Figure 1:**
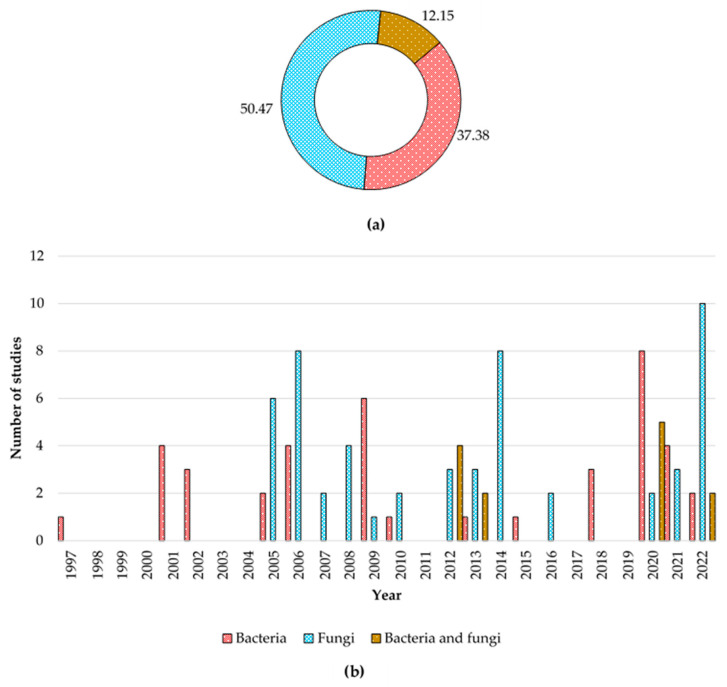
Percentage (**a**) and number of studies (**b**) carried out on coffee endophytes until 2022.

The understanding of endophytes goes back more than a century, in 1866 de Bary introduced the term endophytes for the first time [71]. Later on, discussions were made by further isolation of endophytes from various plant species. In the middle 1930s, Sampson’s [72] and early 1940s, Neill’s [73] studies showed the coexistence of endophyte fungi with, *Lolium temulentum* and *L*. *perenne*. Until the 1960s only a few studies were conducted, thereafter multiple studies have been conducted addressing various dimensions of the endophytes [69]. Understanding the endophytes associated with different plant species also widens [74,75], while the most studied fungal endophytes are the grass associated with the genus *Neotyphodium* (Clavicipitaceae) [76]. According to the literature available, studies on endophytes associated with coffee plants emerged in the later 1990s, Jimenez-Salgado et al. [77] demonstrated the bacterial endophyte *Acetobacter diazotrophicus* associated with coffee. Later on, the next study conducted by Sakiyama et al. [57] isolated several species of *Paenibacillus*. Initial studies focused more on bacterial endophytes; however, fungi endophytes are the most abundant group in the coffee plant rather than the bacteria, and vice versa in coffee-associated soil [78]. According to the literature survey, it is consumable that studies on coffee fungi have sprouted since 2005, and continuously gain more attention than bacterial studies. As shown in Figure 1, several studies only focused on both bacterial and fungal endophytes.

**Figure 2:**
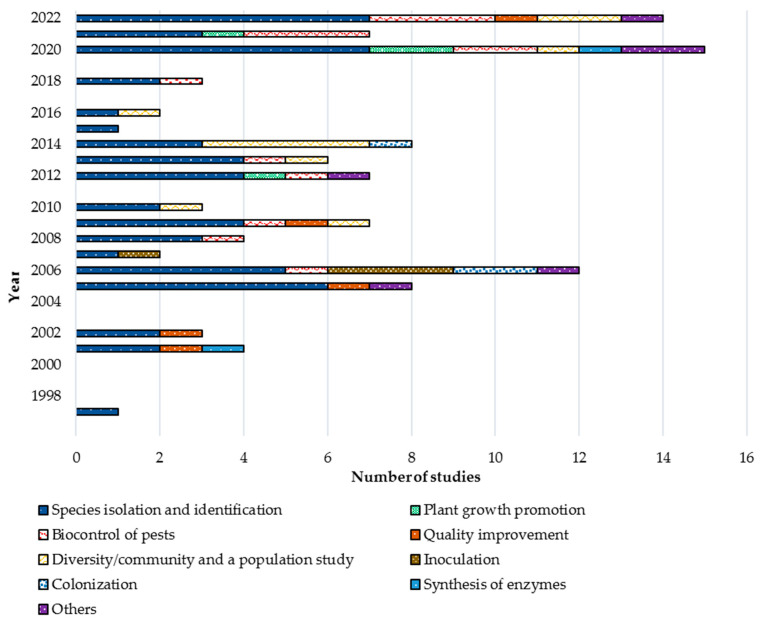
Studies on coffee endophytic fungi. Different dimensions of studies were carried out throughout two and half decades. Most studies focused on isolation and identifying the endophytes associated with coffee plants, followed by biocontrol ability and diversity/population studies. While “other” studies concentrated on endophytic variation, phylogenetic origin, synthesis of mycotoxin, and organic compounds.

Furthermore, just above half of the studies (54.21%) concentrated on isolating and identifying the local coffee endophytes. The next most studies (13.08%) have been carried out to recognize the antagonistic activity of endophytes towards controlling phytopathogens and other pests. A handful of studies (10.28%) are also conducted to understand the in-depth diversity/population of local endophytic microflora. Despite that, several studies revealed the ability of endophytes to improve the quality of coffee seeds (4.67%), coffee plant growth promotion (3.74%), inoculating/introduction to the coffee cultivations (3.74%), and colonization (2.80%). The overview of the carried-out studies is shown in Figure 2.

It is worthwhile to bring some of the study findings on the isolation and identification of coffee endophytes. From 32 isolations of leaf endophytes, Bongiorno et al. [79] observed *Colletotrichum* (71.9%) as the major group, followed by *Trichoderma* (9.4%), *Cercospora* (6.3%) and rest (3.1% each) under the *Ophiognomonia*, *Cladosporium*, *Mycosphaerella*, and *Schizophyllum.* In a comprehensive study, Vega et al. [80] isolated 843 fungal endophytes associated with different coffee plant parts including leaves, roots, stems, and berries, abundant taxa with *Colletotrichum*, *Fusarium*, *Penicillium*, and *Xylariaceae*. Saucedo-García et al. [81] also found similar results, as *Colletotrichum* and *Xylaria* were the most associated endophytes (from 31 morphospecies), nonetheless, even with the coffee leaves. In a similar line, Oliveira et al. [82] identified 17 endophytic fungi species in 16 genera associated with coffee leaves. In a most recent molecular-based study, Gagliardi et al. [83] brought up the fact that the fungi endophytes alone are far more than we think, of course, it is obvious, nevertheless, as a proven study they have come up with 1664 fungal amplicon sequence variants (ASVs) in coffee root tips alone. Table 1 shows some of the endophytes identified to be associated with coffee.

### 3.2. Endophytes in Coffee Plant Growth Promotion 

**Figure 3:**
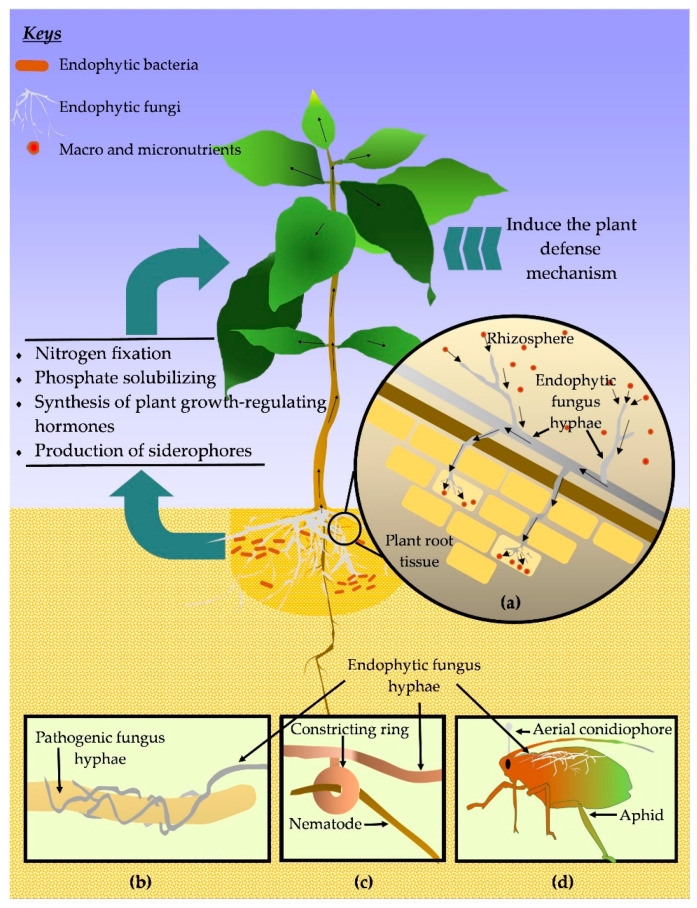
Coffee plant growth improvement and protection mechanism by endophytes. (**a**) Highlight the enhancement of nutrition acquisition by the plant. Hyphae of fungi absorb the nutrients from the rhizosphere and through the invasion/colonization in the plant root system, they supply the carried nutrients to the plant and distribute them throughout the plant. Small arrows show the nutrient movement pathway. (**b**) Endophytic fungi protect plants from other pathogenic fungi through mycoparasitism. (**c**) Some endophytic fungi trap the parasitic nematodes using constricting rings and destroy them. (**d**) Entomopathogenic fungi infect insect pests and spend a parasitic phase inside the living host and infection progress leads the insect to die. The fungi further colonize inside the corpse and further, grow up on the insect body and spend a saprophytic lifestyle.

Plant growth promotion due to endophytes has been widely recognized for various plants including maize [96], potato [97], rice [98], sweet potato [99], tea [100], tomato [101], and so on [102]. It is interesting to know how those coffee endophytes improve plant growth. The most frequent ways that endophytic associations benefit the plants’ growth include direct effects, such as nutrient absorption promotion, enhancing the availability of minerals, nitrogen fixation, secretion of phytohormones, and other bioactive metabolites, indirectly via stress reduction [34,103,104]. The possible pathway of growth promotion in coffee plants is shown in Figure 3.

#### 3.2.1. Nutrient Absorption Promotion and Enhancing the Availability of Minerals

It has been recognized that endophytic fungi have a considerable capacity to improve the nutrient up-taking ability of plants from soil both macro- (e.g., calcium, magnesium, nitrogen, phosphorus, potassium) and micro-elements (e.g., boron, zinc) [105,106]. 

One of the foremost studies conducted by Jimenez-Salgado et al. [77] identified the nitrogen (N) fixing *Acetobacter diazotrophicus* bacteria associated, particularly from the coffee root systems. It has been suggested that increasing populations of endophytic N-fixing bacteria in plants may boost nitrogen fixation, as reported by Jimenez-Salgado and co-authors [77] those recorded bacteria have the ability to colonize extensively, thus increasing values for the plant. In addition, the bacteria found to be in coffee root tissues, Jimenez-Salgado et al. [77] found that they also largely habitat in the rhizosphere. This could be due to the falling of coffee leaves and berries collected in soil lines, degraded by the microbiome, converted further to organic matter, and enriched by the rhizosphere with carbon (sugar) that allows utilization by the *A. diazotrophicus* for their development. By taking into account this phenomenon, it is clear that most probably, N-fixing bacteria are associated with coffee with mutualistic symbiosis, however, no evidence was found during our literature survey. Looking back, Pratiwi et al. [107] isolated several more N-fixing bacteria from coffee roots, among them *Rahnella aquatilis* and *Pseudomonas tolaasii* as the most prominent in fixing N, thus in the ammonium producing. Note that endophytic bacteria modify the molecular nitrogen, then convert it into ammonium, which is then used by host plants for their growth [108]. In turn, further studies conducted by Pratiwi and colleagues [107] recognized that the phosphate (P) solubilizing potentiality of several bacterial species, *Rahnella aquatilis*, and *Kluyvera intermedia* had the highest and most significant P solubilizing capacity. The study findings of them are very competitive with the results obtained by Muleta et al. [109], where they worked on coffee root-associated soil rhizobacterial species, which have P solubilizing capacity. In a recent supportive study, Teshome et al. [110] recognized that *Pseudomonas* spp. (14.5%) are the most prominent P solubilizing in their isolations, followed by *Citrobacter* spp. (3.6%), *Rhodococcus* spp. (9.1%), *Stenotrophomonas* spp. (7.3%), *Gordonia* and *Bacillus* spp. (3.6%). Additionally, Pratiwi et al. [107] recognized that the P solubilizing potentiality of several bacterial species, *Rahnella aquatilis*, and *Kluyvera intermedia* had the highest phosphate solubilizing capacity. 

#### 3.2.2. Secretion of Phytohormones and Other Bioactive Metabolites

It has also been recognized that endophytes synthesize phytohormones (or maybe like compounds), particularly abscisic acid, cytokinin, gibberellins (GAs), and indole-3-acetic acid (IAA), which can promote plant growth development and also aid plant hosts in fending off the negative effects of abiotic stressors [68,111]. In a study, Silva et al. [112] found that bacterial strains of *Acinetobacter calcoaceticus* (161G, 163G, 160G, 150G), *Escherichia fergusonii* (85G), and *Salmonella enterica* (109G) have the ability to synthesize aforesaid IAA, cytokinins, gibberellins, and also siderophores, thus directly enhance the coffee plant growth. Highlighting one such phytohormonal effect, endophytic changes the plant toot architecture by producing plant growth regulators such as IAA, where it affects the roots physiology and function in a positive direction via enhancing its development (also increasing root surface area), leading to better absorption of nutrients and water [105]. While, small molecules known as siderophores are capable of chelating iron, and can be used by plants while depriving pathogens of iron. Further, siderophores aid in fixing nitrogen since diazotrophic organisms need ferrous ions (Fe^2+^) and molybdenum (Mo) factors for their functioning and synthesis of nitrogenase, eventually beneficial to the iron-deficient plant [32]. Recent improvements with technologies, it has been discovered many secondary metabolites secreted by endophytes and their functions [70]. Those molecules not only resistant plants against pests but also protect plants from abiotic stresses to some extent such as drought, flooding, and heat. However, this was not on the agenda relevant to the coffee endophytes particularly how they protect the plants from abiotic challenges; thus, we did not consider those in this paper. 

#### 3.2.3. Successful Field and Laboratory Experiments on Coffee Plant Growth Promotion

**Figure 4:**
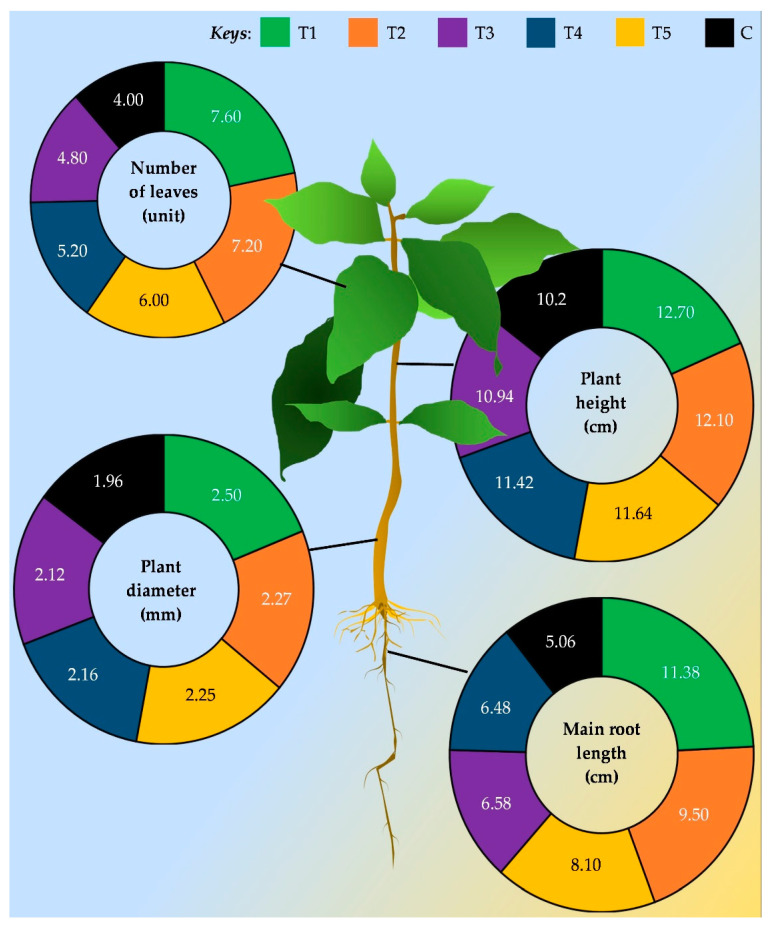
Graphical representation of the data obtained from Mamani-Huayhua et al. [94], which shows the comparison of coffee plant growth parameters along with the *Trichoderma* sp. application. C: Untreated control, T1: Treatment 1; T2–T5: Treatment 2–5. T1–T5 is the application of different fungi strains, TE1, TE2, TE3, TE4, and TE5, respectively.

There are only limited studies conducted on coffee plant growth improvement through endophytes. For instance, Asyiah et al. [113] found that *Bacillus* sp. has the ability to promote plant growth, of this, they have found that *Coffea canephora* grown in pots along with the bacterial consortium increased plant height by an average of 5.2 cm compared with the untreated control. Furthermore, it was observed that the *Bacillus* sp. treatment alone increases the number and area of coffee leaves, e.g., 10.7 ± 2.66 (control: 4.4 ± 2.30) and 416.3 ± 224.9 cm^2^ (control: 109.9 ± 81.5 cm^2^), respectively. Additionally, Mamani-Huayhua et al. [94] showed that *Trichoderma* sp. has a great ability to promote plant growth. They sprayed conidial suspension of 1 × 10^7^ cfu mL^−1^ on soil and foliage at the transplanting of the seedlings, also 30 days intervals after the transplant. They tested the growth parameters of coffee plants after three months of transplanting and, as shown in Figure 4, received considerable evidence for growth enhanced by the means of increases in plant height, stem diameter, length of the main root, and number of leaves compared with untreated control. In an in vitro assay, Silva et al. [111] found that out of 119 bacterial strains, six of them namely, 85G (*Escherichia fergusonii*), 161G, 163G, 160G, 150G (*Acinetobacter calcoaceticus*), and 109G (*Salmonella enterica*) shown significant impact on promoting the growth of coffee seedlings. Further, they have shown that the growth-promoting index for those species is higher as 574, 560, 549, 545, 524, and 522, respectively. In contrast, this study is vital, as they observed, that not all bacterial strains could promote plant growth. In addition, some reduce the growth by having a deleterious effect. However, overall, those findings are clear enough to understand endophytic capacity as growth promoters may be competitive as rhizobacteria. 

### 3.3. Endophytes in Coffee Plant Protection

Endophyte shows the potential to protect plants from a wide range of pathogens and chewing, boring, and sucking insects and nematodes, thus helping them to survive threats [114]. It is intriguing to know first how the endophytes play a role in controlling the phytopathogens. 

#### 3.3.1. Protection against Fungi Pathogens 

It is also interesting to understand the biocontrol mechanism of pathogenic diseases by endophytic bacteria. Secondary metabolites (e.g., harmine), extracellular lytic enzymes (e.g., chitinase, β-1, 3-glucanase, protease, lipase, gelatinases), volatile compounds (e.g., Hydrogen cyanide), and siderophores were discovered to have synergistic effects in inhibiting pathogenic fungus [84,115,116].

Accounting for the endophytic fungi, they inhibit the phytopathogenic fungi using different mechanisms. Those strategies include mycoparasitism, competition, antibiosis, and induction of the plant defense system [117]. In mycoparasitism, the endophytic fungi directly prey on the phytopathogenic fungi. Here, first, the endophytic fungi recognize the praying fungi through the host-based signals and grow towards them, attached, coiling around the prey hyphae, penetrating inside the host, and acquiring the nutrients. This led to the deaths of praying fungi and ultimately controlled the phytopathogenic fungi population [118]. The competition basically takes place among the fungi species for ecological niches and nutrients. Endophytic fungi could grow faster than pathogenic fungi and take advantage of space and nutrients, thus minimizing the colonization of the opponent [54,119]. In antibiosis, to control the pathogens, endophytic fungi use different types of antimicrobial compounds such as aliphatic compounds, alkaloids, peptides, phenylpropanoids, polyketides, and terpenoids [120]. In another way, indirect control of pathogens occurs by inducing the plant defense mechanism. In this regard, along with the association with the plant host, endophytic triggers the induced defense mechanism, by altering the phytohormone biosynthesis, and production of defense-related enzymes and secondary metabolites [121,122,123]. 

Silva et al. [111] found that *Bacillus megaterium*, *Brevibacillus choshinensis*, *Cedecea davisae*, *Microbacterium testaceum*, *Pectobacterium carotovorum*, and *Salmonella enterica* significantly reduces the coffee leaf rust, *Hemileia vastatrix*. Of this, Silva and co-authors [111] recognized that particularly *Brevibacillus choshinensis* shows higher disease-controlling ability where the disease severity index is reduced by 100. Further, it also shows that endophytic bacteria affect the germination of urediniospores. In a similar study, Shiomi et al. [85] conducted a comprehensive study on controlling *Hemileia vastatrix*. First, they isolated the endophytic bacteria from *Coffea arabica* and *Coffea robusta*. They have found that out of 40 selected bacteria isolations, 23 isolations (e.g., *Acinetobacter calcoaceticus*, *Bacillus cereus*, *Bacillus lentimorbus* and *Clavibacter michiganensis*) have the capacity to inhibit *H*. *vastatrix* urediniospore germination in more than 40%. A further compulsive observation was that germination tubes had deformations that impeded normal growth. Furthermore, through the leaf disc assay, Shiomi and co-workers [85] recognized that the endophytic bacteria also have the ability to reduce the disease severity of the coffee leaves caused by *Hemileia vastatrix,* tremendously. In this aspect, *Bacillus lentimorbus* was found to be the most effective, control levels were above 63%. In contrast, Cacefo et al. [124] observed that *H. vastatrix* could also be controlled with *Bacillus subtilis*, however, not as effective as fungicide application where once fungicide showed 53% of disease control, the bacterial treatment could control by 24%. According to the Kejela et al. [116] study, it is clear that *Bacillus* sp. has the ability to control other coffee pathogens such as *Colletotrichum gloeosporioides* and *Fusarium oxysporum*. Even though their study tested under laboratory conditions, the findings are significant; colonies of C. *gloeosporioides* and *F. oxysporum* are inhibited by 78% and 86%, respectively, by *Bacillus* sp. Looking at controlling *Fusarium oxysporum*, Duong et al. [84] recognized that *Burkholderia* sp. (closely related to *B*. *cenocepacia*) shows a higher effect than *Bacillus* sp. (closely related to *B*. *subtilis*), where fungi growth is inhibited by 49.77 ± 0.08% and 40.77 ± 0.06%, respectively. In addition, they found that *Streptomyces* sp. (closely related to *S*. *mobaraensis*) exhibits pathogen controlling ability similar (40.76 ± 0.04%) to *Bacillus* sp. However, a comparatively lower level of *Fusarium oxysporum* inhibition was reported when tested with other genes including *Brachybacterium* sp. (10.26 ± 0.09), *Burkholderia* sp. (26.99 ± 0.01%), *Curtobacterium* sp. (17.68 ± 0.06%), *Kitasatospora* sp. (9.00 ± 0.04%), *Luteibacter* sp. (8.62 ± 0.02%), and *Pseudomonas* sp. (22.18 ± 0.04%).

Coming back to the disease control of coffee leaf rust, endophytic fungi have also proven to be effective as endophytic bacteria. Of this, Mamani-Huayhua et al. [94] presented their findings on controlling *Hemileia vastatrix* using five endophytic *Trichoderma* strains (TE1, TE2, TE3, TE4, TE5). They have sprayed spore suspension, as a coffee foliage application at the laboratory. They found that TE1 and TE2 were significant, whereas TE-1 indicated the best bio-controlling ability as most efficiently reduces disease incidence by 35.8% and severity by 8.95%. Mulaw et al. [89] tested the *Trichoderma* spp. isolated from healthy coffee roots against the coffee tracheomycosis. They have observed that *T*. *flagellatum* strongly inhibits the *Fusarium* spp. In addition, further, they have found that the endophytes control the other pathogens such as *Alternaria alternata*, *Botrytis cinerea*, and *Sclerotinia sclerotiorum*, with inhibition percentages of 39.9 ± 2.2, 38.55 ± 9.56, and 10.67 ± 2.78, respectively. 

In turn, Monteiro et al. [92] demonstrated the effect of volatile compounds produced by coffee endophytic fungi on various phytopathogens. They have found that endophytic *Muscodor coffeanum* (COAD 1842), and *M*. *yucatanensis* (HZM64) completely inhibit the growth of *Botrytis cinerea*, *Cercospora coffeicola*, *Phoma* sp., and *Rizoctonia solani*, while partially inhibit *Aspergillus ochraceus* and *Pestalotia longisetula*. *Muscodor vitigenus* (HZM39) and *Simplicillium* sp. (C12) also showed complete inhibition of *C*. *coffeicola*, *Phoma* sp., and *R*. *solani*, in addition to *P. longisetula*, while partially controlling the *B*. *cinerea*. In another laboratory experiment with *Induratia coffeana*, Gomes et al. [125] showed the antimicrobial activity owing to the volatile compounds produced, against *Aspergillus caespitosus*, *A. elegans*, *A. expansum*, *A. flavus*, *A. foetidus*, *A. niger*, *A. ochraceus A. sclerotiorum*, *A. sydowii*, *A. tamari*, *A. tubingensis*, and *A. versicolor* either by decreasing the growth rate or, mainly, by completely inhibiting colony growth.

Other than the fungal pathogens, bacteria also could cause considerable disease prevalence in the coffee such as bacterial halo blight, bacterial leaf blight, bacterial leaf spot, and coffee leaf scorch; however, the antagonistic effect towards them by endophytes has not yet been tested, which shows another area to cover [126].

#### 3.3.2. Protection against Parasitic Nematodes 

It was found that a number of nematodes also could be associated with the coffee plantations and threaten, e.g., *Apratylenchus* spp., *Criconemella* spp., *Pratylenchus* spp., *Radopholus* spp., *Rotylenchulus* spp., and *Xiphinema* spp. [20]. It is also recorded that endophytic bacteria could control the nematodes, and also use various direct and indirect mechanisms to affect nematodes. For instance, parasitizing, (e.g., producing toxins, antibiotics, extracellular enzymes, and volatile compounds), interfering with nematode–plant-host recognition, competing for nutrients, triggering plant systemic defense mechanisms, and promoting plant health [127]. Enthrallingly, endophytic fungi also control the parasitic nematode by preying on them. In this case, endophytic fungi make constricting rings and trap the nematodes, and absorb the nutrients to let the host die. Those endophytes also have the ability to produce antimicrobial and nematicidal compounds such as linoleic acid or pleurotin [128]. In a greenhouse study, Asyiah et al. [113] planted the Robusta coffee plants with a different bacterial consortium of *Bacillus* sp., and *Pseudomonas dimunita*. They found that the number of *Pratylenchus coffeae* in root or soil on a plant treated with bacterial consortium significantly differed from the control plant. Particularly, the application of *Bacillus* sp. alone indicated 48.2% suppression of the number of *Pratylenchus coffeae* in roots compared to the control. Further, *Bacillus* sp. only also minimizes the nematode population present in the rhizosphere by 54.6%. Contrary to the above findings, Asyiah and co-workers [113] recognized that the combination of endophytic bacteria with other rhizobacteria (e.g., *Pseudomonas dimunita* and *Bacillus subtilis*) showed more effectiveness than the single species isolations. For example, a combination of different endophyte isolates with *Pseudomonas dimunita* suppressed root-inhibiting *Pratylenchus coffeae* by 71.9% and soil dwellings by 76.1%. Note that the beneficial effects of endophytic bacteria on host plants are typically greater than those of rhizospheric bacteria; thus, the importance of endophytic bacteria in the consortium is highly recognized [30]. However, it is quite argumentative to conclude that a consortium of endophytes could work better than single isolation or vice versa. There are several factors that produce varied or inconsistent results due to the following of various study protocols, selected microbial strains, working conditions, geographical variation, and so on [129,130,131].

In one study, Asyiah et al. [87] showed that *Bacillus subtilis* controls and minimizes the penetration of *Pratylenchus coffeae* to coffee plants by up to 85%. A comprehensive study conducted by Hoang et al. [20] found that a coffee root endophytic bacterium, *Streptomyces* sp. has a great ability to control coffee nematode *Meloidogyne incognita*. From an in vitro assay, they observed that nematode eggs and larvae (L2) were destroyed by 85.8% and 85%, respectively. In addition, negative effects on hatched juveniles were also recorded. Intriguingly, Hoang and co-authors [20] reported the further nematicidal activity of *Streptomyces* sp. on nematode cadaver, as the body shrunk after five days and morphologically visible the lysed body just after a week suggesting the toxic mechanism on the host. In a similar study, Mekete et al. [132] recognized several more root endophytic bacterial effects against the *Meloidogyne incognita* (L2 larvae). Further, they have recorded *Agrobacterium radiobacter*, *Bacillus brevis*, *B. licheniformis*, *B. megaterium*, *B. mycoides*, *B. pumilus*, *Cedecea davisae*, *Chryseobacterium balustinum*, *Cytophaga johnsonae*, *Lactobacillus paracasei*, *Micrococcus halobius*, *M. luteus*, *Pseudomonas syringae*, and *Stenotrophomonas maltophilia* sown significant controlling ability of the aforesaid nematode from 38 to 98%. Moreover, Mekete and colleagues [132] observed that *Bacillus pumilus* and *B*. *mycoides* were the most effective in minimizing the number of galls by 33% and egg masses by 39% causing the *M. incognita.* In a similar study, Duong and co-authors [84] reported the lethal effect of *Bacillus* sp. (closely related to *B. cereus* and *B. mycoides*) on *Pratylenchus coffeae* and *Radopholus duriophilus*. 

#### 3.3.3. Protection against Insect Pests

The application of entomopathogenic fungi in controlling pests has become a catchy topic for many researchers [133,134,135]. It is a gripping mechanism to evaluate how entomopathogenic fungi control insect pests. Of this, first fungi spores attach to the cuticle of the insect body, and start to germinate and penetrate through the cuticle (also via the digestive tract) and reach hemocoel. Inside the hemocoel, fungi start to replicate by utilizing nutrients, and insect hosts become weak due to the mycotoxins produced, as well as owing to the tissue invasion and nutrition deprivation, eventually die [136,137,138].

It is possible to find many entomopathogenic fungi naturally habited with coffee plants as endophytes, one such study, for example, Vega et al. [76] identified 16 species of entomopathogenic fungi from five genera, associated with coffee plants, namely *Acremonium* spp., *Beauveria* spp., *Cladosporium* spp., *Clonostachys* sp., and *Paecilomyces* spp. By far, endophytic *Beauveria bassiana* has been the most experimented species, also commercialized worldwide as mycoinsecticides [139]. Owing to their broad range of infecting arthropods, many studies targeted their usage in different crop fields [140,141]. Posada et al. [142] highlighted the different application methods of *Beauveria bassiana* to coffee plants such as foliar spraying, stem injection, and soil drench, which are helpful in introducing to the field when the absence of fungi or improve the field population. In a recent study, Bayman et al. [143] tested 29 local isolations of *B. Bassiana* and also compared their effectiveness with commercially available formulations-Mycotrol in controlling *Hypothenemus hampei*. From the laboratory experiment, they found that the mortality of *H*. *hampei* varied according to the different strains of fungi, and nearly half of the isolations showed over 80% controlling ability. Moreover, several strains (Bb A1, Bb C1, BB M1, N5) were headed the Mycotrol. Further, Bayman and colleagues [143] obtained successful results in their field experiment, which was reported to reduce infestation and the number of pests per fruit. In spite of that, the researchers further highlighted that the effect of *B. bassiana* on the pest depends on the microclimates, or with the changes in the abiotic and biotic environment. According to the study conducted by, Samuels et al. [144], other than *B. bassiana*, *Metarhizium anisopliae* also has the potential to control *H. hampei* significantly, however, may not be greater than *B. bassiana* does. In a subsequent paper, Vega et al. [76] also discussed their findings on managing *H*. *hampei* using *B. bassiana* and *Clonostachys rosea*. According to their results, *B. bassiana* infection leads to the death of insects by just 4.8 ± 0.2 days and 14.7 ± 0.75 days for the insect treated with *C*. *rosea*. 

Jia-ning et al. [145] demonstrated the control ability of coffee stem borers, *Xylotrechus quardripes* and *Acalolepta cervinus* using *Beauveria bassiana* in laboratory and field experiments. They have found effective control of aforesaid pests by *B*. *bassiana* in both environmental conditions. The control ability of different life stages of both pests is recognized, and the mortality percentages of young larvae, elder larvae, pupae, and adult of *X*. *quardripes* after 15 days of fungi application are 98.4 ± 3.2, 96.2 ± 0.5, 94.1 ± 0.8, and 89.1 ± 0.7, respectively. While the mortality percentage of young larvae and elder larvae of *A. cervinus* were 92.4 ± 6.6 and 89.1 ± 5.1, respectively. Further, in the field experiment, the results indicated that coffee stem borer damage was reduced (in 120 days) after applying conidia spray of *B. Bassiana* by 44.4 ± 5.0 and 43.9 ± 4.4 when dusting fungus powder products by hand. 

### 3.4. Role of Endophytes in the Improvement of Quality and Flavor of Coffee Seeds

Microbial communities associated with coffee play a crucial role in determining the quality of the final product. There could be negative and positive effects offered by the microflora, for example, Batista et al. [146] showed that *Aspergillus flavus* and *Penicillium* spp. associated with coffee seeds produce toxic compounds such as aflatoxin (B1 and B2) and ochratoxin A, respectively. Anyway, our perception is of the beneficial effects on endophytes. The importance of endophytes has been shown particularly during the fermentation of coffee beans; however, their role during coffee fruit maturation in natural settings remains unknown [147]. It is believed that the prevalence of certain microbial groups in coffee cherries and the biochemical ramifications of their presence has significantly benefited the quality of coffee by means of improving both aroma and flavor. Pathways of inevitable colonization happening in coffee cherries by endophytes remain uncertain, nevertheless, previously discussed pathways to enter the host could be applicable here, as once endophytes invade, systematic spreading is possible [147]. 

Coming to the point of beneficial effects, the importance of endophytes is recognized during the processing of coffee. First, knowing about coffee fermentation is worthwhile, at this stage; it removes mucilage from parchment coffee. Microorganisms, particularly bacteria, fungi, and yeast play a major role in degrading mucilage by producing various enzymes, alcohols, and acids during the fermentation process. In addition, this step is important for developing aroma and flavor precursors [148]. In an early study, Sakiyama et al. [57] conducted a study with the aim of finding the effect of endophytic bacterial activity on the quality of coffee beverages. They have isolated the *Paenibacillus amylolyticus* associated with coffee beans. Further, Sakiyama and co-authors [57] found that the isolated *P*. *amylolyticus* have the great potential to produce extracellular pectinase, which plays a vital role during fermentation, ultimately enhancing the quality of the beverage. In a recent study, Liu et al. [149] isolated four naturally occurring endophytes namely, *Bacillus cereus* (PMT-1), *Bacillus subtilis* (EDS-1), *Pichia guilliermondii* (EDS-2), and *Priestia aryabhattai* (PMT-2) from the parchment and endosperm of green coffee beans and tested them to understand their ability to improve coffee fermentation. Among them, they have found that *Bacillus cereus*, and *B*. *subtilis* increase the anti-inflammatory chlorogenic acid by 38.5 and 51.5%, respectively, compared to the unfermented beans. 

Today, different levels of caffeine, a stimulant that is the key component in coffee beverages, are favored by consumers, in this regard the importance of coffee endophytes is recognized, above the other traditional methods [150]. Liu and co-workers [149], fermented the coffee with *Pichia guilliermondii* and observed the biodecaffeination ability, dropping caffeine levels by about 38% compared to the unfermented beans. In addition, the study resulted that the aforesaid two *Bacillus* sp. improves the sensory characteristics of the coffee such as sweetness, after-taste, and clean cup. Over the Liu et al. [149] findings on the lower level of biodecaffeination, Baker et al. [87] recognized that *Pseudomonas* sp., exhibiting higher caffeine degradation (98.61%), where cells induce is enough for degrading caffeine as the bacterium uses high concentrated caffeine as a sole source of carbon and nitrogen. Nunes and de Melo. [151] obtained similar results, testing with *Pseudomonas putida* as it completely degraded the alkaloid. In contrast, Chaves et al. [152] recognized that *Aspergillus oryzae* in coffee leaves has the potential to increase caffeine levels. Moreover, Li and colleagues [123] revealed the presence of health-benefiting genes in *B*. *cereus* and *Priestia aryabhattai*, which opened a new window for the research communities. 

## 4. Conclusions

The studies on coffee endophytes are relatively new compared to many other plant-associated microbial studies. In the 25 years of the coffee endophytes’ history of research, only 107 studies have been conducted, basically identifying new species. However, the majority of studies were based on one-time sampling of the plant tissues (mainly, the leaf), whereas periodical sampling seems to be missing. Additionally, many studies were conducted in several geographical locations, making it difficult to comprehend the universal distribution of coffee endophytes. Though there are only a limited number of studies available on both the endophytic ability of coffee plant growth promotion and their applicability as a biocontrol agent, available findings are striking. As most of the studies are limited to lab experiments, there is a necessity to bring them into the field and test them for commercial applicability.

It is also understood that the dynamic of endophytic microflora, due to the climate and other environmental effects, is therefore vulnerable to change in the composition of the population, which may be hindering the actual scenario. Climatic changes, such as drought and flooding, could alter the coffee plant and endophytic interactions. Furthermore, harmful environmental factors, such as pollutants, could change endophytic colonization, and coffee plants may show changes in their usual physiological functions. Up to date, no studies have been conducted in relation to the endophytic effect mitigating the abiotic stress of plants. Thus, future studies could be focused on these as well.

The use of endophytes in coffee farming is undoubtedly advantageous, also by means of avoiding unfriendly pesticides and fertilizers driven for nature-friendly organic farming towards sustainable agriculture. In addition, one fascinating finding would be the use of endophytes in improving the sensory attributes of coffee, thus showing enhanced advantages in the coffee beverage industry. 

## Figures and Tables

**Table 1:** Some of the identified bacterial and fungal coffee endophytes.

Phylum/Division	Species	Associated Coffee Species	Isolated Part of the Coffee Plant	Locality	Reference
**Bacteria**					
**Actinobacteria**	*Arthrobacter* sp.	*C.c* and *C.l*	SE and RO	Buon Ma Thuot, Bao Loc, Vietnam	[84]
	*Clavibacter michiganensis*	*C.c*	LE	State of São Paulo, Brazil	[85]
			SE	Cacahoatán, Chiapas, Mexico	[86]
	*Curtobacterium* sp.	*C.a*	ST	Centro Nacional de Investigaciones de Café, Cenicafé, Chinchin, Colombia	[86]
	*Curtobacterium* sp.	*C.c*	RO	Buon Ma Thuot, Bao Loc	[84]
	*Curtobacterium flaccumfaciens*	*C.a*	SE and LE	Centro Nacional de Investigaciones de Café, Cenicafé, Chinchin, Colombia	[86]
	*Gordona* sp.	*C.a*	CH	Hawaii	[86]
	*Kitasatospora* sp.	*C.c*	RO	Buon Ma Thuot, Bao Loc, Vietnam	[84]
	*Kocuria* sp.	*C.c*	RO	Buon Ma Thuot, Bao Loc, Vietnam	[84]
	*Kocuria kristinae*	*C.a*	LE	State of São Paulo, Brazil	[85]
	*Lechevalieria* sp.	*C.a* and *C.l*	SE and RO	Buon Ma Thuot, Bao Loc, Vietnam	[84]
	*Sinomonas* sp.	*C.c*	RO	Buon Ma Thuot, Bao Loc, Vietnam	[84]
	*Streptomyces* sp.	*C.c* and *C.l*	SE and RO	Buon Ma Thuot, Bao Loc, Vietnam	[84]
**Firmicutes**	*Bacillus* sp.	*C.a*	LE	State of São Paulo, Brazil	[85]
		*C.c* and *C.l*	SE and RO	Buon Ma Thuot, Bao Loc, Vietnam	[84]
	*Bacillus cereus*	*C.c*	LE	State of São Paulo, Brazil	[85]
		*C.a*	LE	Hawaii	[86]
	*Bacillus lentimorbus*	*C.c*	ST	State of São Paulo, Brazil	[85]
	*Bacillus megaterium*	*C.a*	LE	Hawaii	[86]
	*Bacillus subtilis*	*C.a*	CR	Hawaii	[86]
		*C.a*	RO	East Java Province, Indonesia	[87]
	*Lysinibacillus* sp.	*C.c* and *C.l*	SE and RO	Buon Ma Thuot, Bao Loc	[84]
	*Paenibacillus* sp.	*C.c* and *C.l*	SE and RO	Buon Ma Thuot, Bao Loc, Vietnam	[84]
	*Paenibacillus amylolyticus*.	*C.a*	CH	Minas Gerais, Brazil.	[57]
	*Staphylococcus* sp.	*C.c* and *C.l*	SE and RO	Buon Ma Thuot, Bao Loc, Vietnam	[84]
**Proteobacteria**	*Acinetobacter calcoaceticus*	*C.a*	ST	State of São Paulo, Brazil	[85]
	*Acetobacter diazotrophicus*	*C.a*	ST and RO	Guerrero and Puebla, Mexico	[77]
	*Burkholderia* sp.	*C.c*	SE	Buon Ma Thuot, Vietnam	[84]
	*Cedecea davisae*	*C.a*	LE	State of São Paulo, Brazil	[85]
	*Enterobacter* sp.	*C.c*	RO	Buon Ma Thuot, Bao Loc, Vietnam	[84]
	*Herbaspirillum* sp.	*C.c* and *C.l*	SE and RO	Buon Ma Thuot, Bao Loc, Vietnam	[84]
	*Klebsiella pneumonia*	*C.c*	LE	State of São Paulo, Brazil	[85]
	*Luteibacter* sp.	*C.c* and *C.l*	SE and RO	Buon Ma Thuot, Bao Loc	[84]
	*Methylobacterium* sp.	*C.c* and *C.l*	SE and RO	Buon Ma Thuot, Bao Loc, Vietnam	[84]
	*Methylobacterium radiotolerans*	*C.a*	CH	Centro Nacional de Investigaciones de Café, Cenicafé, Chinchin, Colombia	[86]
	*Pandoraea pnomenusa*	*C.a*	LE	State of São Paulo, Brazil	[85]
	*Paracoccus* sp.	*C.c* and *C.l*	SE and RO	Buon Ma Thuot, Bao Loc, Vietnam	[84]
	*Pseudomonas* sp.	*C.a*	ST, RO, and LE (NS)	Southern parts of India.	[88]
**Fungi**					
**Ascomycota**	*Acremonium alternatum*	*C.a*	EP	Caldas, Colombia	[76]
	*Aspergillus* sp.	*C.a*	RO	Ethiopia	[89]
	*Aspergillus oryzae*	*C.a*	LE	Caldas, Colombia	[89]
	*Aureobasidium pullulans*	*C.a*	LE	Pernambuco, Brazil	[82]
	*Beauveria bassiana*	*C.a*	SE, EP, EP, and CR	Caldas, Colombia	[76]
	*Cercospora iranica*	*C.a*	LE	Paraná, Brazil.	[79]
	*Cercospora tezpurensis*	*C.a*	LE	Paraná, Brazil.	[79]
	*Cladosporium* sp.	*C.a*	RO	Ethiopia	[89]
	*Cladosporium cladosporioides*	*C.a*	LE	Adjuntas, Puerto Rico and Kona, Hawaii, USA	[76]
	*Cladosporium* *pini-ponderosae*	*C.a*	LE	Paraná, Brazil.	[79]
	*Cladosporium sphaerospermum*	*C.a*	LE	Beltsville, Maryland, USA	[76]
	*Cladosporium tenuissimum*	*C.a*	LE	Pernambuco, Brazil	[82]
	*Clonostachys rosea*	*C.a*	LE	Chinchina, Caldas, Colombia	[76]
	*Cladosporium cladosporioides*	*C.a*	LE	Pernambuco, Brazil	[82]
	*Colletotrichum asianum*	*C.a*	CH	Chiang Mai, Thailand	[90]
	*Colletotrichum brassicicola*	*C.a*	LE	Veracruz, Mexico	[81]
	*Colletotrichum coffeanum*	*C.a*	LE	Pernambuco, Brazil	[82]
	*Colletotrichum endophytica*	*C.a*	LE	Paraná, Brazil.	[79]
	*Colletotrichum fructicola*	*C.a*	CH	Chiang Mai, Thailand	[90]
	*Colletotrichum gloeosporioides*	*C.a*	LE	Veracruz, Mexico	[81]
	*Colletotrichum gloeosporioides*	*C.a*	LE	Pernambuco, Brazil	[82]
	*Colletotrichum musae*	*C.a*	LE	Veracruz, Mexico	[81]
	*Colletotrichum queenslandicum*	*C.a*	LE	Paraná, Brazil.	[79]
	*Colletotrichum siamense*	*C.a*	CH	Chiang Mai, Thailand	[90]
	*Cryptosporiopsis corticola*	*C.a*	LE	Veracruz, Mexico	[81]
	*Diaporthe liquidambaris*	*C.a*	LE	Pernambuco, Brazil	[82]
	*Dipodascaceae* sp.	*C.a*	RO	Ethiopia	[89]
	*Drechslera biseptata*	*C.a*	LE	Pernambuco, Brazil	[82]
	*Fusarium equiseti*	*C.a*	RO	Ethiopia	[89]
	*Fusarium oxysporum*	*C.a*	RO	Ethiopia	[89]
	*Fusarium solani*	*C.a*	RO	Ethiopia	[89]
	*Glomerella cingulata*	*C.a*	LE	Veracruz, Mexico	[81]
	*Guignardia mangiferae*	*C.a*	LE	Veracruz, Mexico	[81]
	*Induratia coffeana*	*C.a*	NS	Minas Gerais, Brazil	[91]
	*Induratia yucatanensis*	*C.a*	NS	Minas Gerais, Brazil	[91]
	*Khuskia oryzae*	*C.a*	LE	Pernambuco, Brazil	[82]
	*Lasiodiplodia pseudotheobromae*	*C.a*	LE	Pernambuco, Brazil	[82]
	*Macrophomina* sp.	*C.a*	RO	Ethiopia	[89]
	*Muscodor coffeanum* *	*C.a*	LE	Minas Gerais, Brazil	[92]
	Muscodor vitigenus *	*C.a*	ST	Minas Gerais, Brazil	[92]
	Muscodor yucatanensis *	*C.a*	LE	Minas Gerais, Brazil	[92]
	*Mycosphaerella pseudovespa*	*C.a*	LE	Paraná, Brazil.	[79]
	*Nodulisporium gregarium*	*C.a*	LE	Pernambuco, Brazil	[82]
	*Ophioceras leptosporum*	*C.a*	LE	Paraná, Brazil.	[79]
	*Ophiognomonia* sp.	*C.a*	LE	Paraná, Brazil.	[79]
	*Paecilomyces fumosoroseus*	*C.a*	CR	Adjuntas, Puerto Rico	[76]
	*Paecilomyces javanicus*	*C.a*	PE	Chinchina, caldas, Colombia	[76]
	*Paenibacillus amylolyticus*	*C.a*	CH	Minas Gerais, Brazil.	[57]
	*Penicillium* sp.	*C.a*	RO	Ethiopia	[89]
	*Penicillium coffeae*	*C.a*	NS	Kunia, Hawaii	[93]
	*Pestalotiopsis maculans*	*C.a*	LE	Pernambuco, Brazil	[82]
	*Phoma eupyrena*	*C.a*	LE	Pernambuco, Brazil	[82]
	*Phomopsis* sp.	*C.a*	RO	Ethiopia	[89]
	*Phomopsis arnoldiae*	*C.a*	LE	Veracruz, Mexico	[81]
	*Phyllosticta* sp.	*C.a*	LE	Pernambuco, Brazil	[82]
	*Phyllosticta capitalensis* (=*Guignardia mangiferae*)	*C.a*	LE	Pernambuco, Brazil	[82]
	*Pleosporales* sp.	*C.a*	RO	Ethiopia	[89]
	*Sarocladium bacillisporum*	*C.a*	LE	Pernambuco, Brazil	[82]
	*Simplicillium* sp.	*C.a*	ST	Minas Gerais, Brazil	[92]
	*Trichoderma* sp.	*C.a*	LE and ST	San Juan del Oro, Peru	[94]
	*Trichoderma flagellatum*	*C.a*	RO	Ethiopia	[89]
	*Trichoderma hamatum*	*C.a*	RO	Ethiopia	[89]
	*Trichoderma neokoningii*	*C.a*	LE	Paraná, Brazil.	[79]
	*Xylaria* spp.	*C.a*	LE	Veracruz, Mexico	[81]
	*Xylaria* sp.	*C.a*	LE	Pernambuco, Brazil	[82]
**Basidiomycota**	*Rhodotorula acheniorum*	*C.a*	LE	Pernambuco, Brazil	[95]
	*Rhodotorula aurantiaca*	*C.a*	LE	Pernambuco, Brazil	[95]
	*Rhodotorula aurantiaca*	*C.a*	LE	Pernambuco, Brazil	[82]
	*Rhodotorula glutinis*	*C.a*	LE	Pernambuco, Brazil	[95]
	*Rhodotorula mucilaginosa*	*C.a*	LE	Pernambuco, Brazil	[95]
	*Schizophyllum commune*	*C.a*	LE	Paraná, Brazil.	[79]
	*Trametes* sp.	*C.a*	RO	Ethiopia	[89]

*C.a*: *Coffea arabica*; *C.c*: *Coffea canephora*; *C.l*: *Coffea liberica*; CH: Cherrie; CR: Crown; EP: Epicarp; LE: Leaves; NS: Not specified; PE: Peduncle; RO: Roots; SE: Seeds; ST: Stems, * *Muscodor* now transferred to *Induratia* genus.

## Data Availability

Data available on request.

## Supplementary Materials

The following supporting information can be downloaded at: https://www.mdpi.com/article/10.3390/biology12070911/s1, Figure S1: Procedure for the literature selection.
