# Supplementary material for: Coffee-Associated Endophytes: Plant Growth Promotion and Crop Protection
Source: Biology (Basel). 2023 Jun 25;12(7):911. doi: 10.3390/biology12070911 (PMC10376224; doi:10.3390/biology12070911)
Supplement: Supplementary file 1 [file biology-12-00911-s001.zip › biology-2432696-supplementary.pdf]

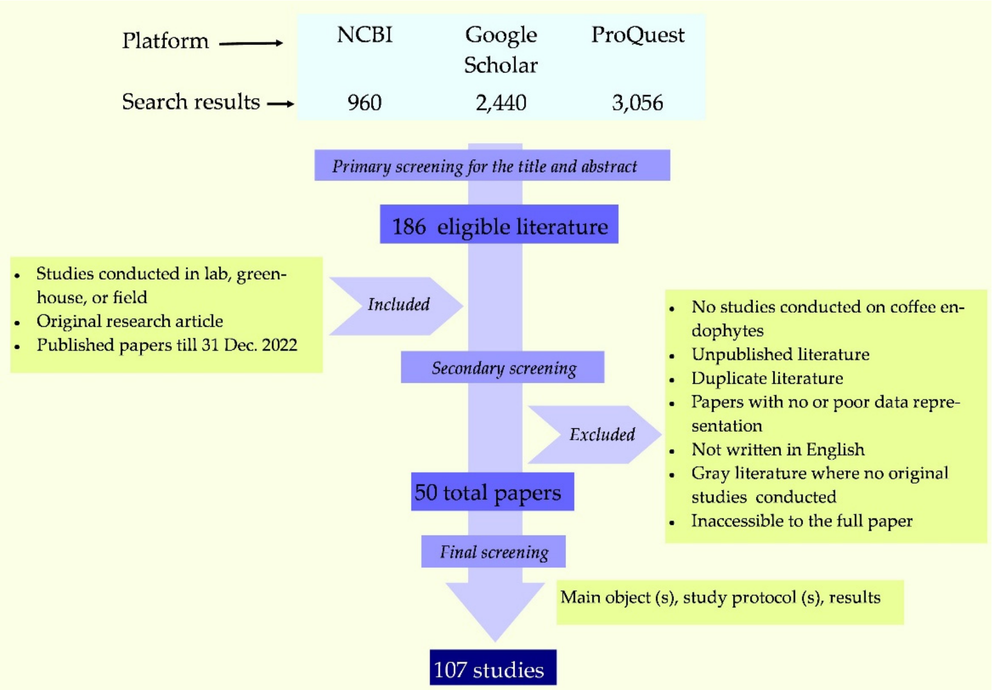

Figure S1. Procedure for the literature selection. A thorough literature search was conducted by using three platforms, namely National Center for Biotechnology Information (NCBI), Google Scholar databases, and ProQuest. The search terminology was, 'Coffee Endophytes', and the initial search resulted in a total of 6,456 findings. However, the majority of results were not directly relevant to our objectives, thus eliminated after the primary screening. Thereafter, extensive secondary screening was done to filter the relevant publication. The final analysis was based on 107 studies from 50 published research papers.
